# Investigation of the anti‐skin aging effects of taurine through mendelian randomization analysis of its relationship with immune cells

**DOI:** 10.1111/jocd.16515

**Published:** 2024-08-20

**Authors:** Hongtao Liu, Honglai Zheng, Siyuan Zhou, Quan Lin

**Affiliations:** ^1^ Guangxi Health Science College Nanning China; ^2^ The Second Affiliated Hospital of Guangxi Medical University Nanning China; ^3^ The People's Hospital of Laibin Laibin China

**Keywords:** anti‐aging treatment, immune cells, Mendelian randomization, monocytes, taurine

## Abstract

**Background:**

Aging skin, exacerbated by external factors like UV radiation and pollutants, is a major cosmetic concern. Taurine, renowned for its antioxidant and anti‐inflammatory properties, may combat skin aging. We performed mendelian randomization (MR) analysis to investigate the causal link between taurine and immune cells linked to skin aging.

**Objectives:**

To investigate the association between taurine and immune cells using mendelian randomization, to thereby explore the mechanism through which taurine exerts anti‐aging effects on the skin via immune modulation.

**Methods:**

A MR approach was employed using taurine‐level data from the Ieu Open GWAS Project and immunocyte traits from a large European cohort. MR‐Egger regression, weighted median estimation, and inverse variance weighting all provided statistical insights into causality. Sensitivity analyses assessed the heterogeneity and pleiotropy among the genetic instruments used.

**Results:**

MR analysis identified a causal relationship between taurine levels and 10 immunocyte phenotypes, with taurine found to be negatively and positively associated with three and seven phenotypes, respectively. Sensitivity analysis revealed no significant heterogeneity or pleiotropy, suggesting reliable MR findings.

**Conclusion:**

This study provides insights into the immunological pathways by which taurine contributes to skin anti‐aging effects, suggesting that increasing taurine levels could offer a novel strategy for anti‐aging skincare.

## 1 INTRODUCTION

Anti‐aging of the skin is a crucial aim of many cosmetic treatments, as it can help improve the appearance and texture of the skin, slow down the skin aging process, and contribute to the restoration of skin health and youthfulness. Skin aging is significantly influenced by a variety of external factors, including ultraviolet radiation (UVR), particulate matter (PM), and the microbiome, which disrupt the skin immune barrier and accelerate skin aging.^1^ Further studies have suggested that skin aging is associated with numerous immune cells, among which T‐cells and mononuclear macrophages play crucial roles.^2^

Taurine is an amino acid that exerts multiple physiological effects and potential health benefits. Its functions include maintaining normal tissue function, promoting fat digestion and absorption, regulating blood glucose levels, and exerting antioxidant and anti‐inflammatory properties.^3^ Taurine exists in high concentrations in the skin and plays a role in maintaining skin homeostasis.^4^ Meanwhile, taurine plays a significant role in defending skin inflammation in the human body. Animal experiments have confirmed that taurine can prevent skin inflammation induced by UVR by promoting the expression of Nrf2‐mediated antioxidant/anti‐inflammatory enzymes.^5^ One study recently published in the journal “Science”^6^ confirmed the longevity‐enhancing effects of taurine through animal experimentation. This study has sparked significant interest in the anti‐aging properties of taurine. However, this research was conducted solely in animal models, and the actual health benefits of taurine in humans therefore require confirmation through randomized controlled trials (RCTs).

Mendelian randomization (MR) is a methodology used to assess causality in observational studies. This method is based on Mendelian inheritance principles, which suggest that the inheritance of genetic variation occurs randomly. Consequently, genetic variations associated with specific diseases may influence the variables under consideration. MR thus investigates the correlation between exposure and outcomes by contrasting populations with distinct genetic variations. This approach provides superior control over potential confounding factors compared to conventional observational studies, thereby enhancing the reliability of the study conclusions.^7^ In summary, the advantage of MR studies lies in their utilization of naturally randomly assigned genotypes to infer causal relationships between biological factors and diseases, effectively eliminating the interference of confounding factors and enhancing the accuracy and reliability of causal inference.

This study aimed to investigate the relationship between taurine levels and immune cells using MR analysis, and to explore the mechanism by which taurine improves skin aging through immune modulation. It is anticipated that this research will provide a novel avenue for anti‐aging treatment of the skin.

## 2 MATERIALS AND METHODS

### 2.1 Acquired data

We acquired taurine level data [ID: ebi‐a‐GCST90026035, Year:2021, Population: European, Sex: NA, *n*: 291, Number of SNPs: 6856779] from the Ieu Open GWAS Project (http://gwas.mrcieu.ac.uk), a genome‐wide association studies database.^8^ Immunocyte data were obtained as outcomes using the same method. Identification numbers for the data ranged from GCST0001391 to GCST0002121. This study comprised a genome‐wide association analysis of 629 blood immune cell‐related traits in 272 100 individuals from the general European population, including 731 immunophenotypes.^9^

#### 2.1.1 Mendelian randomization analysis

Following data acquisition, we conducted separate evaluations for two associations: (1) between SNPs and exposure, and (2) between each SNP and outcome. Subsequently, MR analysis was performed to evaluate the causal association between taurine levels and each immunophenotype.

Three statistical methods were used to investigate the relationship between taurine levels and immunophenotypes: MR‐Egger regression, weighted median estimation, and inverse variance weighting (IVW). MR‐Egger regression, which is employed in MR studies, is a statistical method used to detect and account for pleiotropic effects. Pleiotropy refers to the phenomenon in which a genetic variant influences outcomes through multiple pathways. The MR‐Egger approach incorporates instrumental variables to estimate the causal effect of exposure on an outcome, while accounting for potential pleiotropic effects.^10^ The weighted median estimator is a statistical technique used to estimate the representative value of a population by calculating the median score of a weighted sample. In this method, individual data points in a sample are assigned weights that reflect their relative importance or influence on the overall estimation of population parameters. This approach is particularly advantageous when dealing with outliers in data distribution, as it offers a more robust measure of central tendency than conventional methods such as the arithmetic mean.^11^ IVW is a statistical methodology frequently used in meta‐analyses to combine effect size estimates obtained from multiple studies. The weight assigned to each study in IVW was determined based on the inverse of its variance, which represented the precision of the effect estimate. Considering both the sample size and variability in effect sizes across studies, the IVW approach generates a pooled effect size incorporating information from the entire body of evidence.^10^

To ensure the accuracy of MR analysis, it is crucial that the genetic variation of the chosen instrumental variables exhibits a strong correlation with the risk factors. Therefore, we set the significance level at *p* < 5 × 10^−8^ as the criterion for screening instrumental variables.^9^, ^12^

### 2.2 Sensitivity analysis

The IVW method was applied to calculate the *Q*‐value^13^ to assess the heterogeneity between SNPs, and the *Q*_*p‐*value was calculated to determine heterogeneity. Furthermore, we conducted a “leave‐one‐out” analysis to investigate the potential influence of individual SNPs on the causal association. Finally, we applied MR‐Egger regression tests to monitor the presence of potential horizontal pleiotropy effects.

### 2.3 Statistical method

MR analysis was conducted using the “TwoSampleMR” package in R (V4.2.3).^14^ Calculation of the causal association between taurine levels and each immunophenotype was reported as an odds ratio (OR) with a corresponding 95% confidence interval (CI). The threshold for statistical significance was set at *p* < 0.05.

## 3 RESULTS

### 3.1 Mendelian randomization analysis

The MR results are presented in Tables 1 and 2, with a threshold of *p* < 0.05 defining a causal relationship. When the IVW method yielded significance (*p* < 0.05), even if the other methods did not, a positive result can be considered, provided that the beta values of the other methods align in the same direction.^15^ Based on these results, 10 immunocyte phenotypes associated with taurine levels were identified. Among these, four immunocyte phenotypes were negatively associated with taurine levels (Table 1). In contrast, the remaining six immunocyte phenotypes were positively associated with the taurine levels (Table 2). The details of each SNP are presented in TableS1.

**TABLE 1:** Results of Mendelian randomization of four immune cell phenotypes negatively associated with taurine levels.

ID	Immune cell phenotype	Method	nsnp	b	SE	pval	OR	95% CI		*Q*_*p*‐value (heterogeneity)	Egger_intercept	SE	*p*‐Value (pleiotropy)
ebi‐a‐GCST90001529	CD66b^++^ myeloid cell absolute count	MR‐Egger	12	−0.201	1.226	0.873	0.818	0.074	9.038	0.143	−0.029	0.062	0.655
		Weighted median	12	−0.803	0.422	0.057	0.448	0.196	1.023				
		IVW	12	−0.741	0.343	0.031	0.477	0.243	0.933	0.181			
ebi‐a‐GCST90001781	CD25 on IgD^+^ CD38‐ naive B cell	MR‐Egger	12	−0.931	1.003	0.375	0.394	0.055	2.813	0.466	0.014	0.051	0.789
		Weighted median	12	0.394	0.055	2.813	0.467	0.208	1.049				
		IVW	12	−0.669	0.301	0.026	0.512	0.284	0.923	0.549			
ebi‐a‐GCST90001927	CD127 on CD8^+^ T cell	MR‐Egger	12	−1.490	0.770	0.082	0.225	0.050	1.020	0.800	0.054	0.039	0.199
		Weighted median	12	−0.302	0.311	0.332	0.740	0.402	1.361				
		IVW	12	−0.478	0.228	0.036	0.620	0.397	0.969	0.707			
ebi‐a‐GCST90001916	CD45 on CD4^+^ T cell	MR‐Egger	12	−0.189	0.776	0.813	0.828	0.181	3.789	0.434	−0.020	0.040	0.626
		Weighted median	12	−0.449	0.321	0.162	0.638	0.340	1.198				
		IVW	12	−0.561	0.231	0.015	0.571	0.363	0.897	0.501			

**TABLE 2:** Results of Mendelian randomization of 6 immune cell phenotypes positive associated with taurine levels.

ID	Immune cell phenotype	Method	nsnp	b	se	*p*‐Value	OR	95% CI		*Q*_*p*‐value (heterogeneity)	Egger_intercept	SE	*p*‐Value (pleiotropy)
ebi‐a‐GCST90001888	CD28 on CD39^+^ secreting CD4 regulatory T cell	MR‐Egger	12	1.257	0.795	0.145	3.513	0.740	16.687	0.532	−0.038	0.041	0.370
		Weighted median	12	0.562	0.330	0.089	1.755	0.918	3.353				
		IVW	12	0.544	0.236	0.021	1.722	1.086	2.733	0.541			
ebi‐a‐GCST90001967	FSC‐A on CD14^+^ monocyte	MR‐Egger	12	0.430	0.886	0.638	1.538	0.271	8.737	0.256	0.009	0.045	0.854
		Weighted median	12	0.503	0.351	0.152	1.654	0.831	3.292				
		IVW	12	0.590	0.252	0.019	1.804	1.100	2.959	0.328			
ebi‐a‐GCST90001969	FSC‐A on Natural Killer	MR‐Egger	12	0.924	0.865	0.311	2.519	0.462	13.727	0.281	−0.019	0.044	0.678
		Weighted median	12	0.540	0.332	0.104	1.715	0.895	3.289				
		IVW	12	0.571	0.248	0.021	1.770	1.088	2.878	0.343			
ebi‐a‐GCST90001975	FSC‐A on HLA DR^+^ T cell	MR‐Egger	12	0.944	0.775	0.251	2.569	0.562	11.740	0.942	−0.022	0.040	0.592
		Weighted median	12	0.565	0.304	0.063	1.760	0.970	3.193				
		IVW	12	0.534	0.231	0.021	1.706	1.085	2.682	0.956			
ebi‐a‐GCST90002034	CD39 on monocyte	MR‐Egger	12	1.763	0.824	0.058	5.828	1.159	29.309	0.861	−0.068	0.042	0.138
		Weighted median	12	0.494	0.349	0.156	1.639	0.828	3.247				
		IVW	12	0.495	0.244	0.043	1.641	1.016	2.649	0.712			
ebi‐a‐GCST90002074	SSC‐A on CD14^+^ monocyte	MR‐Egger	12	0.777	0.801	0.355	2.174	0.452	10.459	0.405	−0.017	0.041	0.695
		Weighted median	12	0.429	0.313	0.171	1.536	0.831	2.838				
		IVW	12	0.468	0.235	0.046	1.598	1.009	2.530	0.479			

### 3.2 Sensitivity analysis

**FIGURE 1:**
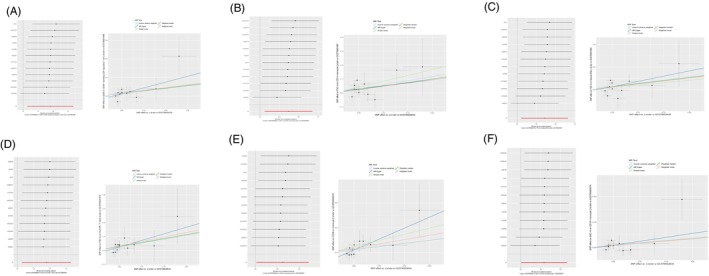
Sensitive analysis of positively correlated immune cell phenotypes, (A) ebi‐a‐GCST90001888, (B) ebi‐a‐GCST90001967, (C) ebi‐a‐GCST90001969, (D) ebi‐a‐GCST90001975, (E) ebi‐a‐GCST90002034, (F) ebi‐a‐GCST90002074.

**FIGURE 2:**
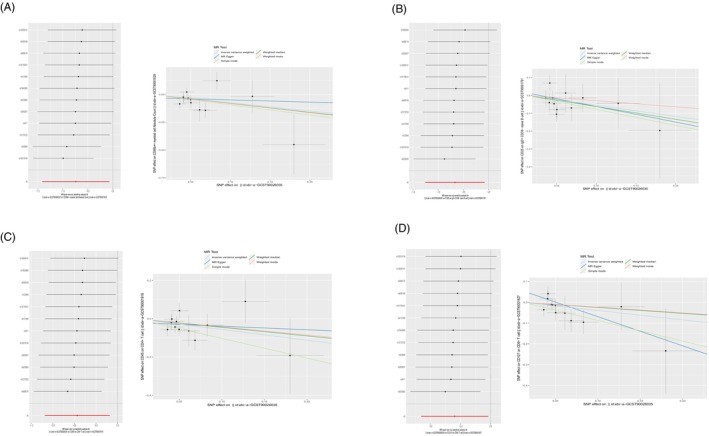
Sensitive analysis of negative correlated immune cell phenotypes, (A) ebi‐a‐GCST90001529, (B) ebi‐a‐GCST90001781, (C) ebi‐a‐GCST90001916, (D) ebi‐a‐GCST90001927.

The results of the heterogeneity tests indicated no heterogeneity among the SNPs in all MR Analyses (*Q*_*p* > 0.05) (Tables 1 and 2). In this study, we conducted all MR analyses using the “leave‐one‐out” approach. After the sequential removal of each SNP, the direction of the beta values calculated using the IVW method remained consistent across all analyses (Figure 2); this indicates the stability of all MR findings.^16^ Furthermore, we subjected all intercept terms (egger_intercept) of the MR‐Egger method to statistical testing, and the resulting *p*‐values were >0.05. Therefore, we concluded that there was no evidence of horizontal pleiotropy (Figures 1 and 2).^17^

## 4 DISCUSSION

The skin acts as the outermost barrier organ of the human body, providing both a physical and an immune defense. As an active immune‐protective organ, the skin contains various types of immune cells, including mononuclear phagocytes (MNP), Langerhans cells (LC), dendritic cells, macrophages, monocytes, and T cells.^2^ With increasing age, functional skin immune surveillance gradually decreases, leading to increased susceptibility to skin infections and cancer; skin infections may also accelerate skin aging.^18^

### 4.1 The role of T cells as immune sentinels in skin immunity

T cells circulate between the skin‐draining lymph nodes and peripheral tissues to mount rapid responses to antigenic challenges.^19^, ^20^ They further act as the first line of defense against various bacterial and fungal skin.^21^ This study identified a positive correlation between three T‐cell phenotypes and taurine levels. CD28, a co‐stimulatory receptor, plays a crucial role in the activation and function of regulatory T cells (Tregs). On the surface of CD4^+^ T cells, CD28 engagement by its ligands, such as CD80 and CD86, provides a second signal required for T cell activation and effector function. CD39, an ectonucleotidase, is expressed by a subset of Tregs, and promotes immune tolerance by converting extracellular ATP into adenosine, which inhibits pro‐inflammatory immune responses.^22^ The expression level of CD28 on CD39^+^–secreting CD4 regulatory T cells determines the extent of immune activation^23^; as such, the appropriate addition of taurine is conducive to immune activation.

HLA DR^+^ T cells play critical roles in immune regulation, antimicrobial infection, and tumor immunity. These cells present antigens and activate other immune cells.^24^ Forward scatter area (FSC‐A) is a parameter used in flow cytometry to measure cell size.^25^ A larger FSC‐A value may indicate that HLA‐DR^+^ T cells possess larger size or granularity characteristics. This may be associated with activation status, proliferative capacity, or cell subtype. A higher FSC‐A value suggests that the HLA‐DR^+^ T cells are in an activated state, or belong to a functionally specialized subset. As such, our findings demonstrate that taurine can activate HLA DR^+^ T cells.

FoxP3 and CD127 are important immunological markers. FoxP3 is expressed in Tregs, and thus serves as a marker of this subpopulation.^26^ CD127 is primarily present on the surface of CD8^+^ T cells, and is an important indicator of their function and survival capacity.^27^

Research has shown that the development and function of Tregs require the expression of FoxP3, which can regulate immune responses by suppressing the activation of other T cell subsets. Conversely, the function and survival capacity of CD8^+^ T cells are primarily associated with the expression level of CD127. CD8^+^ T cells with high CD127 expression are typically classified as memory T cells that exhibit enhanced functionality and protective abilities.

In various disease states such as autoimmune diseases and tumors, the quantity and functionality of Tregs and CD8^+^ T cells can undergo alterations. For example, an increase in the number of Treg cells may lead to immune tolerance, whereas the downregulation of CD127 expression in CD8^+^ T cells can contribute to immune responses.^28^ Therefore, our results demonstrated that elevated taurine levels can effectively reduce the expression of CD127, thereby achieving the desired immune response.

### 4.2 The role of monocytes in skin immunomodulation

CD14^+^ monocytes, a specific subset of white blood cells that express the CD14 protein on their surfaces, are an important cell type in the immune system. CD14 is a receptor involved in cell‐mediated immune responses. CD14^+^ monocytes play critical roles in immune responses, inflammatory reactions, and antimicrobial activity. They are capable of recognizing and binding bacterial components, and participate in immune defense and regulation by activating inflammatory mediators, producing cytokines, and promoting the activation of other immune cells. CD14^+^ monocytes can also differentiate into macrophages or dendritic cells, further participating in the immune response.^29^

Monocytes play crucial roles in skin immunomodulation. As a form of white blood cells, monocytes can differentiate into several types of immune cells, including dendritic cells and macrophages. Upon recruitment to the skin, monocytes perform several functions. First, they help defend the skin by phagocytosing and eliminating harmful pathogens. Second, they present processed antigens derived from pathogens on their cell surfaces, thereby triggering the activation of T cells and initiating an immune response. Additionally, monocytes secrete cytokines that regulate the immune response of skin immune cells. Finally, they can differentiate into tissue‐specific cells such as fibroblasts, and contribute to the repair of damaged skin tissues. Monocytes play an indispensable role in regulating skin immunity and preventing various skin diseases.^30^, ^31^, ^32^

In general, a higher FSC‐A value indicates a larger cell size, whereas a lower value indicates a smaller cell size. In CD14^+^ monocytes, a larger cell size may be associated with the activation status or other cellular properties.^25^

The side scatter intensity (SSC‐A) parameter of CD14^+^ monocytes is used to evaluate the granularity or internal complexity of the cells. In general, larger SSC‐A values are associated with increased cell granularity or complexity, which may indicate that the cells are in an activated state, or possess specific functional features.^25^

In summary, based on our findings, it can be inferred that higher levels of taurine activate the immunoreactivity of CD14^+^ monocytes, as manifested by increased values of SSC‐A and FSC‐A. Therefore, the role of NK cells in skin immunity should not be overlooked. The skin serves as the outermost protective barrier of the body, and is constantly exposed to various pathogens and environmental stressors. NK cells, a subset of specialized lymphocytes, possess both natural cytotoxicity and immunoregulatory functions. In the context of skin immunity, NK cells directly eliminate abnormal cells from the skin, secrete cytokines to modulate the activity of other immune cells, and establish memory immune responses. These properties enable NK cells to protect the skin. Additionally, other immune cells such as dendritic cells and macrophages interact with NK cells, highlighting the importance of their collaborative regulation in maintaining skin health, defending against skin diseases, and providing potential therapeutic interventions.^33^, ^34^, ^35^ These findings suggest that elevated taurine levels can enhance FSC‐A expression in Natural Killer cells, indicating that high taurine levels may enhance skin defense mechanisms.

In summary, our results showed that increasing taurine levels can enhance immune activity by regulating immune cells such as T cells and monocytes. Although taurine is considered a relatively safe nutritional substance, excessive taurine intake may lead to gastrointestinal discomfort, such as bloating, diarrhea, nausea, and vomiting. It can also negatively affect blood pressure and metabolism.^36^ Therefore, further clinical research is needed to determine the appropriate levels of taurine required to achieve safe anti‐aging effects on the skin.

This study has certain limitations. First, there were limitations in sample size and ethnic diversity in the GWAS database. Second, MR analysis cannot analyze dose–response relationships; therefore, further clinical observations and experimental studies are warranted. The subjects of this study are Europeans, who have relatively low taurine intake due to dietary habits and other reasons.^37^ Therefore, more extensive ethnic studies are needed to verify our results.

## 5 CONCLUSION

Overall, this study elucidated the immunological mechanism by which taurine combats skin aging using MR analysis of the relationship between taurine levels and immune cells. Overall, our results indicate that appropriately increasing taurine levels can exert anti‐aging effects on the skin. It is well known that exercise and taurine intake can elevate taurine levels in the body, which could provide a novel avenue for anti‐aging skin treatments.

## FUNDING INFORMATION

The 2023 Special Research Project for high‐level talents of Guangxi Health Science College (No. GXWZY202301).

## CONFLICT OF INTEREST STATEMENT

The authors declare no conflicts of interest.

## ETHICS STATEMENT

Public database was used in this study.

## Supporting information


Table S1.

## Data Availability

The data that support the findings of this study are available in gwas at http://gwas.mrcieu.ac.uk, reference number ID: ebi‐a‐GCST90026035. These data were derived from the following resources available in the public domain: gwas, http://gwas.mrcieu.ac.uk
